# Perspectives on the Genomics of HSP Beyond Mendelian Inheritance

**DOI:** 10.3389/fneur.2018.00958

**Published:** 2018-11-26

**Authors:** Dana M. Bis-Brewer, Stephan Züchner

**Affiliations:** ^1^Dr. John T. Macdonald Foundation Department of Human Genetics, University of Miami Miller School of Medicine, Miami, FL, United States; ^2^John P. Hussman Institute for Human Genomics, University of Miami Miller School of Medicine, Miami, FL, United States

**Keywords:** heredit spastic paraplegia, genomics, risk allele, Mendelian, non-Mendelian inheritance

## Abstract

Hereditary Spastic Paraplegia is an extraordinarily heterogeneous disease caused by over 50 Mendelian genes. Recent applications of next-generation sequencing, large scale data analysis, and data sharing/matchmaking, have discovered a quickly expanding set of additional HSP genes. Since most recently discovered HSP genes are rare causes of the disease, there is a growing concern of a persisting diagnostic gap, estimated at 30–40%, and even higher for sporadic cases. This missing heritability may not be fully closed by classic Mendelian mutations in protein coding genes. Here we show strategies and published examples of broadening areas of attention for Mendelian and non-Mendelian causes of HSP. We suggest a more inclusive perspective on the potential final architecture of HSP genomics. Efforts to narrow the heritability gap will ultimately lead to more precise and comprehensive genetic diagnoses, which is the starting point for emerging, highly specific gene therapies.

## Introduction

Hereditary spastic paraplegias (HSPs) are a genetically heterogeneous group of neurodegenerative disorders with a prevalence of ~3-9/100,000 in most populations and a clinical hallmark of progressive lower limb weakness and spasticity (1, 2). HSPs result from genetic alterations resulting in dysfunction of the long axons in the corticospinal tract and posterior columns (3). Consistent with the cardinal clinical features of HSP, the primary pathological mechanism is distal axonal degeneration in a “dying-back” manner (4). HSPs are broadly categorized into pure and complicated forms based on the presence of additional clinical features such as ataxia, parkinsonism, peripheral neuropathy, cognitive dysfunction, cataracts, and icthyosis (3). HSPs segregate in several modes of inheritance, including autosomal dominant, autosomal recessive, X-linked, or mitochondrial (3). Although there is no evidence that HSP overall is more prevalent in one ethnic group over another, HSP does show ethnic differences in many of the mutated genes (Table 1).

**Table 1:** Critical review of the current SPG loci curated from the Online Mendelian Inheritance in Man (OMIM) database and a PubMed literature review of “hereditary spastic paraplegia” and each HSP gene and/or loci number.

**Designation**	**Locus**	**Gene**	**Predominant phenotype**	**Inheritance**	**OMIM number**	**Year first reported**	**Publication count**	**Family count**	**Ethnicity**	**PubMed ID**
SPG1	Xq28	L1CAM	Complicated	X-linked	303350	1986	25+	–	–	–
SPG2	Xq22.2	PLP1	Complicated	X-linked	312920	1987	20+	–	–	–
SPG3A	14q22.1	ATL1	Pure	AD	182600	1987	100+	–	–	–
SPG4	2p22.3	SPAST	Pure	AD	182601	1994	200+	–	–	–
SPG5A	8q12.3	CYP7B1	Pure	AR	270800	1994	20+	–	–	–
SPG6	15q11.2	NIPA1	Pure	AD	600363	1995	12	14	Irish; Iraqi; Chinese; Japanese; Brazilian; Hungary	7825577; 14508710; 25341883; 21599812; 21419568; 17928003; 16795073; 16267846; 15643603; 27084228; 25133278; 24075313
SPG7	16q24	SPG7	Pure or complicated	AR and AD	607259	1998	60+	–	–	–
SPG8	8q24.13	WASHC5	Pure	AD	603563	1999	8	19	Caucasian; Brazilian; British; European; Dutch; Chinese; Japanese	9973294; 10797436; 17160902; 23455931; 29768361; 27957547; 26967522; 25454649
SPG9	10q23.3-q24.1	ALDH18A1	Complicated	AD	601162	1999	4	9	Italian; French; Spanish; Portuguese; Australian; Japanese	9973297; 26026163; 26297558; 29915212
SPG10	12q13.3	KIF5A	Pure	AD	604187	1999	40+	–	–	–
SPG11	15q21.1	SPG11	Complicated	AR	604360	1999	100+	–	–	–
SPG12	19q13.32	RTN2	Pure	AD	604805	2000	3	4	Welsh	10677333; 22232211; 27165006
SPG13	2q33.1	HSPD1	Pure	AD	605280	2000	3	2	French; Dutch	10677329; 11898127; 17420924
SPG14	3q27-q28	SPG14	Complicated	AR	605229	2000	1	1	Italian	10877981
SPG15	14q24.1	ZFYVE26	Complicated	AR	270700	2001	11	31	Arab; Irish; Tunisian; French; Austrian; Italian	11342696; 17661097; 19438933; 18394578; 19805727; 27217339; 26492578; 24833714; 23733235; 19084844
SPG16	Xq11.2	SPG16	Complicated	X-linked	300266	1997	2	2	Japanese; NA	9254866; 10982474
SPG17	11q12.3	BSCL2	Complicated	AD	270685	2001	9	23	Austrian; English; Brazilian; Italian; Chinese; Portuguese; Korean; Dutch	11389484; 13680364; 14981520; 29934652; 25487175; 25219579; 18612770; 17486577; 16427281
SPG18	8p1123	ERLIN2	Complicated	AR and AD	611225	2011	6	7	Turkish; Arabian; Northern European; Chinese; Saudi Arabian	21330303; 23109145; 29528531; 27824013; 23085305; 21796390
SPG19	9q	–	Pure	AD	607152	2002	1	1	–	12112072
SPG20	13q13.3	SPART	Complicated	AR	275900	2002	7	8	Amish; Omani; Turkish; Filipino; Moroccan	12134148; 18413476; 20437587; 26003402; 27112432; 28679690; 27539578
SPG21	15q22.31	ACP33	Complicated	AR	248900	2003	2	2	Amish; Japanese	14564668; 24451228
SPG23	1q24-q32	DSTYK	Complicated	AR	270750	1985	3	3	Middle Eastern	4061404; 14681889; 28157540
SPG24	13q14	–	Pure	AR	607584	2002	1	1	Saudi Arabian	12499481
SPG25	6q23-q24.1	–	Complicated	AR	608220	2002	1	1	Italian	12070243
SPG26	12p11.1-q14	B4GALNT1	Complicated	AR	609195	2013	2	7	Spanish; Portuguese; Tunisian; Bedouin	23746551; 24283893
SPG27	10q22.1-q24.1	–	Complicated	AR	609041	2004	1	1	French Canadian	15455396
SPG28	14q22.1	DDHD1	Pure	AR	609340	2005	3	4	Moroccan; Turkish; French; Japanese	23176821; 15786464; 27216551
SPG29	1p31.1-p21.1	–	Complicated	AD	609727	2005	1	1	Scottish	16130112
SPG30	2q37.3	KIF1A	Pure	AR and AD	610357	2011	11	17	Palestinian; Algerian; Saudi Arabian; Brazilian; Macedonian; Korean; Sicilian; Chinese	21487076; 22258533; 25585697; 26410750; 27034427; 28332297; 28362824; 28834584; 28970574; 29159194; 29934652
SPG31	2p11	REEP1	Pure	AD	609139	2006	30+	–	–	–
SPG32	14q12-q21	–	Complicated	AR	611252	2007	1	1	Portuguese	17515546
SPG33	10q24.2	ZFYVE27	Pure	AD	610244	2006	1	1	German	16826525
SPG34	Xq24-q25	–	Pure	X-linked	300750	2008	1	1	Brazilian	18463901
SPG35	16q23.1	FA2H	Complicated	AR	612319	2008	14	28	Arab; Omani; Pakistani; Filipino; Chinese; Italian; Turkish	19068277; 20104589; 20853438; 22965561; 23566484; 23745665; 24359114; 24833714; 26344562; 27217339; 27316240; 28017243; 29376581; 29423566
SPG36	12q23-q24	–	Complicated	AD	613096	2009	1	1	German	19357379
SPG37	8p21.1-q13.3	SPG37	Pure	AD	611945	2008	1	1	French	17605047
SPG38	4p16-p15	SPG38	Complicated	AD	612335	2008	1	1	Italian	18401025
SPG39	19p13.2	PNPLA6	Complicated	AR	612020	2008	5	10	Ashkenazi Jewish; German; Japanese; Brazilian	18313024; 24355708; 23733235; 25631098; 29248984
SPG41	11p14.1-p11.2	SPG41	Pure	AD	613364	2008	1	1	Chinese	18364116
SPG42	3q25.31	SLC33A1	Pure	AD	612539	2008	1	1	Chinese	19061983
SPG43	19q12	C19ORF12	Complicated	AR	615043	2013	1	1	Malian	23857908
SPG44	1q42.13	GJC2	Complicated	AR	613206	2009	1	1	Italian	19056803
SPG45	10q24.3-q25.1	NT5C2	Complicated	AR	613162	2009	3	2	Turkish; Qatari	19415352; 24482476; 28327087
SPG46	9p13.3	GBA2	Complicated	AR	614409	2013	6	12	Tunisian; Cypriot; Chinese	23332916; 23332917; 24252062; 29524657; 27553021; 24337409
SPG47	1p13.2	AP4B1	Complicated	AR	614066	2011	7	13	Israeli; Arab; Turkish; African American	24781758; 22290197; 24700674; 24781758; 29193663; 27625858; 25693842
SPG48	7p22.1	AP5Z1	Pure	AR	613647	2010	3	4	French; Moroccan	20613862; 24833714; 26085577
SPG49	14q32.31	TECPR2	Complicated	AR	615031	2012	3	3	Jewish Bukharian	23176824; 25590979; 27406698
SPG50	7q22.1	AP4M1	Complicated	AR	612936	2009	4	5	Moroccan; Turkish; Greek	19559397; 21937992; 24700674; 29096665
SPG51	15q21.2	AP4E1	Complicated	AR	613744	2011	4	4	Palestinian; Syrian; Moroccan	20972249; 21620353; 21937992; 23472171
SPG52	14q12	AP4S1	Complicated	AR	614067	2011	3	5	Syrian; Caucasian; Albanian; Italian	21620353; 25552650; 27444738
SPG53	8p22	VPS37A	Complicated	AR	614898	2012	1	2	Arab	22717650
SPG54	8p11.23	DDHD2	Complicated	AR	615033	2012	4	9	Dutch; Canadian; Omani; Iranian	23176823; 23486545; 24482476; 27679996
SPG55	12q24.31	C12ORF65	Complicated	AR	615035	2012	3	3	Japanese; Indian	24080142; 24198383; 23188110
SPG56	4q25	CYP2U1	Complicated	AR	615030	2012	6	9	Saudi Arabian; Southern Italian; Iranian; Japanese; Turkish; Italian	23176821; 24337409; 26936192; 27292318; 28725025; 29034544
SPG57	3q12.2	TFG	Complicated	AR	615658	2013	5	6	Indian; Italian; Pakistan; Sudanese; British Pakistani; northern Indian	23479643; 29971521; 28124177; 27601211; 27492651
SPG58	17p13.2	KIF1C	Complicated	AR and AD	–	2014	3	6	Palestinian; Moroccan; Turkish	24319291; 24482476; 29544888
SPG61	16p12.3	ARL6IP1	Complicated	AR	615685	2014	1	1	–	24482476
SPG62	10q24.3	ERLIN1	Complicated	AR	615681	2014	1	3	–	24482476
SPG63	1p13.3	AMPD2	Complicated	AR	615686	2014	1	1	–	24482476
SPG64	10q24.1	ENTPD1	Complicated	AR	615683	2014	1	2	–	24482476
SPG72	5q31.2	REEP2	Pure	AR/AD	615625	2014	2	3	French; Portuguese; –	24388663;28491902
SPG73	19q13.33	CPT1C	Pure	AD	616282	2015	1	1	–	25751282
SPG74	1q42.1	IBA57	Complicated	AR	616451	2015	1	1	Arab	25609768
SPG75	19q13.1	MAG	Complicated	AR	616680	2014	2	2	Pakistani; –	26179919; 24482476
SPG76	11q13.1	CAPN1	Complicated	AR	616907	2016	4	9	Moroccan; Utah; Irish; Italian	27153400; 29678961; 27320912; 28566166
SPG77	6p25.1	FARS2	Pure	AR	617046	2015	3	4	Chinese; European	25851414; 26553276; 29126765
SPG78	1p36.1	ATP13A2	Complicated	AR	617225	2016	2	4	Pakistani; Bulgarian	27217339; 28137957
SPG79	4p13	UCHL1	Complicated	AR	615491	2013	3	3	Turkish; Norwegian; Indian	23359680; 28007905; 29735986

As with many other Mendelian diseases, the introduction of next-generation sequencing (NGS) revolutionized the genetic diagnosis of HSPs with over 76 genomic loci and 58 corresponding genes (5). The autosomal dominant (AD) HSPs lead mostly to the pure form of disease and are linked to 19 spastic gait (SPG) genes. The most common AD genes are SPG4/*SPAST*, SPG3A/*ATL1*, SPG31/*REEP1*, and SPG10/*KIF5A*. Complicated HSPs more frequently occur in autosomal recessive (AR) families and are linked to 57 loci and 52 genes. The most common AR genes are SPG11/*KIAA1840*, SPG5A/*CYP7B1*, SPG7, and SPG15/*ZFYVE26*. Though rare, X-linked and mitochondrial inheritance are also observed. In the past 5 years alone >15 novel HSP and HSP-related genes have been reported. However, the number of families identified in the initial and follow-on papers is typically low; for example, the original *REEP2* publication had 2 families with a follow-on of a single family (6, 7). Furthermore, a large number of recent SPG loci are supported by a single publication only (Table 1). This has led to the concern that we are in an asymptotic situation where, even with many new genes, the diagnostic yield may not get close to 100%. This potential gap in heritability is observed in other rare disorders as well and might be referred to as “dark matter” of clinical genomics. This review will focus on potential causes and efforts to overcome these challenges.

## The “dark matter” of clinical genomics

It is usually assumed that HSP is caused by Mendelian mechanisms and that eventually nearly all patients will receive a single-gene diagnosis. However, this is not necessarily true. Related disorders illustrate a diverse situation: inherited neuropathies (Charcot-Marie-Tooth disease, CMT) are also highly heterogeneous Mendelian disorders whereas amyotrophic lateral sclerosis (ALS) is largely not explained by Mendelian genes. The proportion of Mendelian genes is even lower in late onset neurodegenerative diseases, such as Parkinson and Alzheimer disease.

The reported diagnostic yield for exome sequencing in the general clinical setting ranges from 25 to 50% (8, 9). In hereditary spastic paraplegias, Schüle et al. identified a molecular diagnosis in 46% of families (240/519 families), despite extensive whole-exome sequencing efforts, with low success in simplex families (28%) (2). Similarly, a 20% diagnostic yield was reported in a cohort of 98 previously unsolved HSP families analyzed by a custom sequencing panel of 70 HSP and HSP-related genes (10).

**Figure 1:**
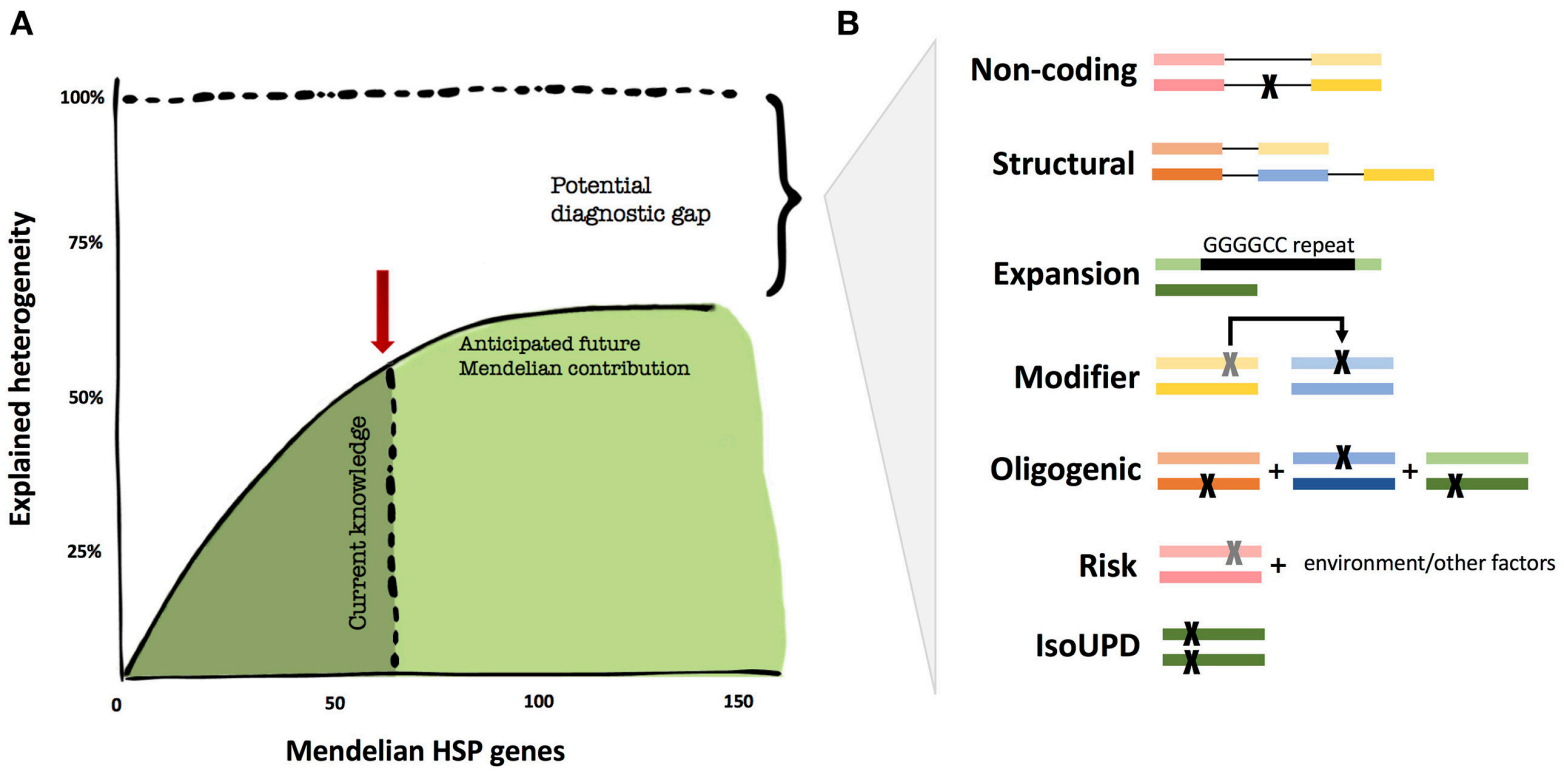
The diagnostic heritability gap in HSP. **(A)** Despite unprecedented success in the identification of additional Mendelian genes, the diagnostic yield may not get close to 100% in HSP, but rather reach an asymptotic ceiling. **(B)** Areas that are potentially understudied in HSP thus far for cost and technical challenges. These include uncommon Mendelian causation, but also modifier and oligogenic risk alleles. The colored bars represent genes, the black lines connecting genes represent noncoding regions, and each “X” represents a mutation.

While the genetics of certain neurodegenerative diseases are deemed “complex,” the field, thus far, has not applied similar models of inheritance to HSP. We believe that it is too early to tell whether non-Mendelian effects have a major contribution to HSP. There are a number of valid pro-Mendelian hypotheses as to how to close the diagnostic gap, and the coming years will allow us to test these ideas (Figure 1). These include non-coding regions of the genome, unorthodox types of mutations (such as repeat expansions) and digenic inheritance models. However, the search for risk genes and alleles will likely contribute to the understanding of HSP in multiple ways, from exploring oligogenic causation, gene/environment interactions, and phenotype modifying genes; thus, expanding the inheritance models in HSP.

## The continued search for mendelian causes

Systematic reanalysis of unresolved clinical exomes can reveal causative variants that were not prioritized in the initial analysis and increase the diagnostic yield; however, the search should ideally be expanded beyond mutations in the protein-coding regions (11). Full exploration of the non-coding space will require whole-genome sequencing; however, fortunately, the untranslated regions and regions adjacent to exons are typically covered by whole-exome sequencing. Though sparse annotation poses considerable challenges in interpreting these variants, Minnerop et al. found deep intronic mutations in *POLR3A* to be a frequent cause of HSP and cerebellar ataxia. After identifying intronic variants in a single recessive spastic ataxia family, the authors screened a cohort of 618 cases. They found that compound heterozygous *POLR3A* mutations accounted for ~3.1% of genetically unclassified autosomal recessive and sporadic index cases. Over 80% of these cases shared the same deep-intronic mutation which activates a cryptic splice site. This study nicely demonstrates the potential held within the non-coding genome.

As the limitation of non-coding coverage is overcome by whole-genome sequencing, the clinical and functional interpretation of variants will remain a challenge. Recently, the complementation of genetic sequencing with transcriptome sequencing (RNA-seq) has successfully improved the diagnostic yield in Mendelian disorders (12, 13). RNA-seq adds a functional layer to help prioritize genes and variants by providing information about aberrant splicing, abnormal gene expression levels, and extreme cases of mono-allelic expression (13). Since gene expression and mRNA isoforms vary widely across tissues, sequencing disease-relevant tissue is critical to the potential value with this approach (12). Due to the difficulties in obtaining the disease-relevant tissue, transcriptome sequencing may have limited potential in HSP. However, the recent mapping of the axonal transcriptome from induced pluripotent stem cell derived motor neurons may provide an avenue to overcoming this challenge (14).

Another contributor of genome variability that could help resolve the diagnostic gap is structural variation, including copy number variations, translocations, and inversions (15). Copy number variations (CNVs), in particular, are known to play an important role in HSPs, causing 8%-41% of SPG4 due to the *Alu* genomic architecture of *SPAST* (16–19). Screening of approximately 600 independent HSP cases over a 5-year period in *ATL1, SPAST, NIPA1, SPG7*, and *REEP1* has detected numerous deletions (20). Recently, a complex homozygous 4-kb deletion/20-bp insertion that removes the last two exons and part of the 3′UTR was identified in *DSTYK* (21). These studies exemplify the relevance of structural variation in HSPs. However, major challenges still exist that hinder full exploration of these mutations. Since CNVs are also a large source of normal variation and their pathogenic potential can depend on genetic ancestry and environmental factors, determining whether a CNV is benign or pathogenic remains a considerable clinical challenge (15, 22). Additionally, CNV detection from whole-exome sequencing remains immature and unstable, as indicated by the low concordance between CNV variant callers (23). Another popular method for CNV detection is high-resolution microarray, which is commonly used in clinical cytogenetics where sequencing based analysis can overwhelm a typical laboratory's workflow (24). Though research laboratories largely rely on NGS, the additional high-resolution microarrays may lead to diagnoses in cases with suspected CNV. Improved sequencing technology and structural variation interpretation and detection will resolve more HSP families in the future.

## Underappreciated rare genomic mechanisms

Standard clinical genomic analysis focuses on typical modes of inheritance, such as autosomal dominant, autosomal recessive, and X-linked, while unusual inheritance modes are often ignored. Inclusion of these “genomic” mechanisms in analysis pipelines, for example genomic imprinting, repeat expansions, and uniparental disomy, can lead to successful identification of overlooked molecular diagnoses.

Uniparental isodisomy is particularly relevant to HSPs as this mechanism has been observed in two autosomal recessive spastic ataxia of Charlevoix-Saguenay (ARSACS) cases, one SPG18 case, and four SPG35 cases (25–27). Uniparental isodisomy is the inheritance of both chromosomes from the same parent, resulting from errors in meiosis and/or mitosis (28). Isodisomic events generate regions of homozygosity in the offspring, allowing for the inheritance of a homozygous variant from one heterozygous carrier-parent (29). During trios analysis, these homozygous variants are usually discarded as sequencing errors since only one parent is a carrier. The occurrence of uniparental isodisomy can be inferred from very long regions of homozygosity that are isolated to a single chromosome; therefore, uniparental isodisomy detection can be incorporated into a standard whole-exome pipeline by modifying existing methods for long regions of homozygosity (30, 31). The high number of SPG35 cases resulting from UPD suggests that this may be a frequent genetic mechanism for *FA2H* homozygous mutations in non-consanguineous families (27). Uniparental isodisomy is important to detect because of its impact on molecular diagnosis and recurrence risk in families.

The awareness of the genetic overlap between HSPs and spinocerebellar ataxias (SCAs) has increased as the number of loci causing both diseases expands; however, the overlap of HSP and SCA caused by triplet-repeat expansions is less emphasized (32). Bettencourt et al. reviewed the literature of triplet-repeat expansions mimicking spastic paraplegias: expansions have been observed at the *ATXN1, ATXN2, ATXN3, ATXN7, ATN1*, and *FXN* loci (32). The authors recommend incorporating triplet-repeat ataxia analysis into diagnostic algorithms, especially *ATXN3* in autosomal dominant complicated HSP and *FXN* in autosomal recessive or sporadic spastic paraplegias (32). It is conceivable that additional, yet to be identified, coding and non-coding repeat expansion loci cause HSP.

## Beyond mendelian inheritance

Based on the assumption of fully penetrant alleles, traditional Mendelian disease analysis focuses on the rare DNA variation that segregates within a family. However, these locus-specific family studies treat Mendelian traits as distinct entities and disregard a more comprehensive genetic model for human disease in which variants of varying effect size as well as environmental influences contribute to disease (33). The challenge is the unexpected large amount of variation in the human genome on a population level, where >99% of all variants show a minor allele frequency of < 1% (34). Since many of these variants are without phenotypic consequence, some certainly are very harmful, and a considerable number must have effect sizes that are below the threshold of a Mendelian gene but contribute significantly to phenotypic expression. Identification of strong effect sizes in the background of mostly minor effects is the next big challenge in human genetics. Recent method developments in statistical genetics allow for unbiased genome-wide screens for non-Mendelian alleles, and surprisingly, are able to re-identify *bona fide* Mendelian genes as well (35). The application to HSP genomics will eventually generate a more complete genetic architecture of the disease.

### Reduced penetrant and risk alleles

Contrary to general expectations for HSP families, asymptomatic carriers are not infrequent, in which case, the genotype is said to be incompletely penetrant (36, 37). Reduced and age-dependent penetrance is a diagnostically challenging situation observed in autosomal dominant HSPs, especially SPG3A, which can lead to misinterpretation of inheritance patterns due to asymptomatic carriers and exclusion of the disease-causing allele (38–40). Additionally, sex-dependent penetrance is suspected in *SPAST* and *ATL1* based on the excess of affected males (41). Incomplete penetrance can also manifest in autosomal recessive disorders when the primary mutation leads to varying phenotypic effects depending on the secondary mutation (36). For example, novel compound heterozygous mutations in *SPG11* let to an atypical late onset and mild form of SPG11 (42).

To distinguish reduced-penetrance alleles, which have caused HSP in at least some individuals under a Mendelian trait, risk alleles have been defined as variants with smaller effect sizes that are part of a multifactorial model of disease causation (36). However, since the possibility of risk alleles is only recently recognized in rare Mendelian disease, the line between penetrance and risk is often blurred. In this context, risk alleles more broadly refer to rare variants that may lead to a less severe, later-onset form of disease or contribute to an individual's susceptibility to disease, likely through an oligogenic model. For example, heterozygous mutations in *SPG7* were identified as a potential susceptibility factor for late-onset neurodegenerative disorder (43). Similarly, heterozygous mutations in *MME* were recently shown to predispose carriers to late-onset axonal neuropathy (44). In MME, the comparison of the “rare variant load” of missense and loss of function changes in late-onset CMT to the general population showed a significant enrichment of such variation (44).

Systematic identification of rare variant associations are usually limited by low statistical power unless sample sizes or variant effect sizes are very large (45). To illustrate, >60,000 cases (and an equal number of controls) would be necessary to detect a disease association for a rare variant (0.1% frequency) with an odds ratio of 2.0 for a disease with a 5% population prevalence (45). Fortunately, powerful study designs can alleviate the sample size requirement to more reasonable numbers (46). One approach that can be explored in HSPs is the gene-based variant burden test which collapses the number of minor alleles into one genetic score (gene), thus reducing multiple testing and increasing power (45, 47). One successful example of this approach was the identification of a new ALS gene, *TBK1*, in 2,869 sporadic ALS patients (35). Remarkably, other known ALS genes showed strong associations, indicating that additional variation in known familial ALS genes also contribute to sporadic ALS forms (35). The rare variant association studies are particularly useful for identifying risk genes and novel gene associations.

### Modifier alleles

An increasing number of exceptions to the fundamental “one gene, one phenotype” paradigm are being published across Mendelian phenotypes (48). The oversimplified view that phenotypic expression, even for classically monogenic disorders, is driven exclusively by mutations at a single locus is being replaced by the concept of genetic modification (49). Though several types of genetic modification are possible, the simple definition is the effect of one allele on the phenotypic outcome of a second allele (49). If the primary allele is sufficient to cause disease, then the secondary allele is a “modifier” that modulates phenotypic expression, such as disease severity or progression.

Given the high clinical variability observed across HSP patients, genetic modification of the primary allele was anticipated. Over a decade ago, intragenic polymorphisms were suggested to modify the age at onset of *SPAST* mutations (50, 51). More recently, *SPAST* deletions spanning the adjacent *DPY30* gene were shown to have significantly reduced age at onset (52). Furthermore, in a study of a large Cuban spinocerebellar ataxia type 2 (SCA2) cohort, 33% of the residual age at onset variance was attributed to genetic modifiers (53). Examples of genetic modifiers from related Mendelian disorders exist in the literature; for instance, a polymorphism in miR-149 was recently associated with onset age and severity in Charcot-Marie-Tooth disease type 1A (CMT1A) (54).

Another study design that increases the statistical power for association testing of rare variants is the extreme phenotype sampling (EPS) approach (46). Based on the assumption that rare causal variants are more likely found in the extremes of a quantitative trait such as age of onset or severity of a symptom, EPS can increase the power to detect rare variants over random sampling (46). For example, Emond et al. utilized an extreme phenotype sampling approach to identify an association between rare coding variants in *DCTN4* and time to first *Pseudomonas* infection (measure of cystic fibrosis severity) (47, 55). Additionally, Tao et al. identified *SIPA1L2* as a genetic modifier of muscle strength impairment in CMT1A. *In vitro* knock down of *SIPA1L2* in Schwannoma cells lead to a significant reduction in *PMP22* expression, offering a potential pathway for therapeutic strategies (56). Application of EPS to an HSP cohort may also reveal modifier alleles that contribute to disease.

### Oligogenic inheritance

Digenic or oligogenic inheritance refers to instances when one primary allele is insufficient to cause disease, instead requiring the combined consequence of multiple alleles (49). Evidence of oligogenic inheritance has emerged in other neurological disorders. In both sporadic and familial amyotrophic lateral sclerosis (ALS) cases, patients harboring two or more rare variants had lower survival or earlier age at onset, suggesting that the combined effect of rare variants affects ALS development and progression (57–59). Similarly, over 30% of Parkinson's disease (PD) patients carried additional rare variants in Mendelian PD genes and had younger ages at onset (60). An increased rare variant burden was also observed in two cohorts of inherited neuropathy cases, which was followed up *in vivo* zebrafish experiments (61). In zebrafish, more severe phenotypic outcomes were observed as a consequence of increased mutational burden in neuropathy genes, consistent with a positive genetic interaction mechanism of oligogenic inheritance (61).

Demonstrating oligogenic inheritance from family studies is challenging without experimental models. However, one trending approach to assessing oligogenic inheritance—which has been explored in Parkinson, ALS, Frontotemporal Dementia, Congenital Hypothyroidism, Inherited Neuropathy, and more—is to evaluate the mutational burden across known disease genes through Fisher's exact test or logistic regression (60–64). However, caution should be used with this approach as Koegh et al. warns that systematic bias can lead to the apparent enrichment of “oligogenic” variants in familial cases and controlling such bias is essential for investigating an oligogenic role in neurodegenerative diseases (65).

## Connecting the many loci: a network biology approach

Though the high amount of locus heterogeneity present in HSPs complicates clinical diagnosis, it does provide an opportunity to study the overarching biological pathways through analysis of molecular networks. Genes and their products form complex networks within cells that are governed by specific laws and principles (66). These complex networks model the non-linear genotype-phenotype relationships observed in HSPs, such as incomplete penetrance and epistasis, that deviate from the “one gene, one phenotype” principle (66, 67). Functionally related proteins interact with each other to accomplish similar biological mechanisms, thus forming cellular pathways (66). Network medicine capitalizes on these interactions and hypothesizes that perturbation of a single gene product will propagate along the entire network (68). The interactions between a set of disease-causing genes can be summarized into a disease module for further study (69). A disease module can be analyzed for differences in complete loss of gene products vs. interaction-specific perturbations, global relationships to other human diseases, novel candidate disease genes, and emerging biological pathways (68, 70–73). Novarino et al. combined exome sequencing with network analysis to summarize a global view of HSP (74). A HSPome was created from previously published HSP seed genes and candidate genes from whole exome sequencing. From the HSPome, they extracted subnetworks of functionally related proteins that form pathological modules, including ER-associated degradation, endosomal and membrane-trafficking, and purine nucleotide metabolism. Three candidate genes arose from the HSPome that were found to be mutated in HSP patients. Lastly, the authors discovered the HSP seed genes were significantly overlapping with amyotrophic lateral sclerosis, Alzheimer's disease, and Parkinson's disease, while no overlap was observed between HSP and neurodevelopmental disorders nor non-neurological disorders (74). Recently, the relationship between the inherited axonopathies, HSP and Charcot-Marie-Tooth 2 (CMT2), was explored through a network analysis of protein-protein interactions. The HSP disease module was found to significantly overlap both the CMT2 and hereditary ataxia modules. Pathway analysis revealed ribosomal protein and viral infection response pathways (75). With the rapid pace of gene discovery in HSPs, network analysis will continue to be a powerful approach for deciphering the complex interactions underlying the phenotype.

## Overcoming the phenotypic divide

Historically, many movement disorders, including HSPs, have been clinicogenetically classified based on the predominant phenotype of the first gene locus (76). These classification systems have similar shortcomings, including erroneously assigned loci, duplicated loci, missing loci, and unconfirmed loci (76, 77). Furthermore, these classification systems suggest that HSPs are a distinct and isolated disorder, when in fact HSPs exist on a spectrum between inherited ataxias and axonal Charcot-Marie-Tooth disease (CMT2) (76, 78). Not only do these disorders share clinical symptoms, such as prominent lower extremity spasticity, but they can also be caused by mutations within the same genes (76, 78, 79). Next-generation sequencing greatly facilitated the appreciation of these genetic overlaps by providing an unbiased approach that broke through the prior clinical and diagnostic preconceptions (76). Phenotypic expansions continue to blur the lines drawn in neurological disorders; for example, HSP was recently associated to *PLA2G6*, the causative gene underlying heterogeneous PLA2G6-associated neurodegeneration (PLAN) (80). Additionally, *KIF5A*, known to cause both HSP and CMT2, contains a C-terminal hotspot of mutations that can cause a classical amyotrophic lateral sclerosis phenotype (81). Furthermore, mutations in *ATP13A2*, originally linked to a rare form of juvenile-onset atypical Parkinson disease (Kufor-Rakeb syndrome), are now also associated with neurodegeneration with brain iron accumulation, neuronal ceroid lipofuscinosis, and most recently, complicated HSP (82–84). Awareness and consideration of phenotypic expansions will be essential for both individual genetic diagnoses as well as revealing common pathways underlying neurodegeneration (80). As the phenotypic spectrum broadens across the neurologic community, these historical classifications are being reconsidered (78). To address this issue, Synofzik and Schüle have proposed a mechanism based classification system for the ataxia-spasticity spectrum, based on unbiased modular phenotyping, that captures nuanced phenotypic expression, opens ataxia and spasticity to a multisystem neuronal dysfunction, and help to prioritize research on shared pathways (76).

## The necessity of data aggregation and collaboration

The above-mentioned approaches increasingly require larger datasets which contradicts, of course, the low prevalence of rare disease. This requirement exceeds what single labs have traditionally been able to collect from local clinics. It will be insufficient to exchange candidate gene information or candidate alleles. To apply statistical approaches, one needs to gather hundred if not thousands of HSP samples and adequate controls. This led to the notion of raw genetic data aggregation as the next frontier for HSP gene discovery. The most prominent example of systematic data aggregate in HSP is the GENESIS Project database (tgp-foundation.org). Over 600 HSP, 500 ataxia, and 890 CMT exomes or genomes have been aggregated from several dozen laboratories in 22 different countries(85). We have begun to complement Mendelian gene discovery efforts with modifier gene studies and rare variant burden analyses. It is still early, but it appears that significant results can be achieved beginning with 200+ exomes and sufficient control samples. For example, an exome-wide association study with 202 cases and 6,905 controls successfully found a signal in *GREB1L* (joint *p*-value = 2.3 × 10^−7^) in renal agenesis and hypodysplasia (86). Another exome-wide association study in CMT, with 343 cases and 935 controls, identified a significant association in *EXOC4* (*p*-value = 6.9 × 10^−6^, OR = 2.1) and nominal associations with other known CMT genes (87).

## Concluding remarks

The current period is a remarkable time for HSP research. Studies from many countries are reporting a steady pace of novel Mendelian genes, complementing existing multigene clinical panels. The diagnostic yield has never been higher; albeit it is hindered by an increasing burden of Variants of Uncertain Significance. In addition, gene therapy approaches are maturing in related disorders, and it appears only a matter of time before they are applied to specific HSP genes. These novel therapeutic approaches include gene replacement, antisense oligonucleotides (ASO), and soon gene editing. Most of them require a specific genetic diagnosis. This emphasizes the need to fill the diagnostic gap currently estimated at 30-40%, but larger for sporadic HSP cases.

Contemplating the details of recently discovered HSP genes suggests that traditional Mendelian mutations may not be able to account for the majority of yet to be diagnosed patients. We have outlined the various possibilities of non-protein coding Mendelian variation but also an increasing interest in multigene causation or phenotypic modification. It appears to soon to tell how the genetic architecture of HSP will look like in 10 years' time. However, we are certain that non-Mendelian elements will play a role, if only as secondary protective/worsening factors. Still, cohort approaches now enabled through extensive collaborations and data aggregations will likely also hold surprises for non-traditional disease causation in HSP.

## Author contributions

All authors listed have made a substantial, direct and intellectual contribution to the work, and approved it for publication.

### Conflict of interest statement

The authors declare that the research was conducted in the absence of any commercial or financial relationships that could be construed as a potential conflict of interest.
